# Subcutaneous and visceral adipose tissue in patients with primary and recurrent incisional hernia

**DOI:** 10.1007/s10029-021-02416-6

**Published:** 2021-04-22

**Authors:** H. Qandeel, C. Chew, D. Young, P. J. O’Dwyer

**Affiliations:** 1 Department of Surgery, Hashemite University, Zarqa, Jordan; 2 Department of Radiology, University Hospital Hairmyres, Glasgow, UK; 3 Department of Mathematics and Statistics, Strathclyde University, Glasgow, UK; 4 School of Medicine, Dentistry and Medicine, University of Glasgow, Glasgow, UK

**Keywords:** Obesity, Primary incisional hernia, Recurrent incisional hernia, Adipose tissue, Incisional hernia

## Abstract

**Purpose:**

Visceral obesity rather than body mass index has been reported to be associated with a higher incidence of incisional hernias. The aim of this study was to examine the relationship between CT measured adipose tissue and muscle in primary and recurrent incisional hernia.

**Methods:**

Patients with a ‘Primary’ or ‘Recurrent incisional hernia’ were obtained from a prospective cohort of patients who were being assessed for incisional hernia repair over a 2-year period. Computerised tomography (CT)-images were analysed using NIH Image-J software to quantify adipose tissue and skeletal muscle cross-sectional areas at the level of lumber vertebra 3/4 using standard Hounsfield units. To test inter-observer ‘absolute agreement’, each parameter was measured independently by two investigators and reliability analysis performed.

**Results:**

Thirty-six patients were included in the study: 15 had a *Primary* while 21 had a *Recurrent* incisional hernia. Both groups had similar baseline characteristics. Reliability analysis for CT-measured areas showed very high interclass correlation coefficient (ICC) between observers. Patients in the recurrent group had significantly greater subcutaneous adipose tissue (SAT) [median = 321.9cm^2^ vs 230.9cm^2^, *p* = 0.04] and visceral adipose tissue (VAT) [median = 221.1cm^2^ vs 146.8cm^2^, *p* = 0.03] than those in the primary group. There was no difference in skeletal muscle areas for right [median = 2.8cm^2^ vs 2.9cm^2^] and left [median = 3.7cm^2^ vs 4.1cm^2^] rectus muscles between groups.

**Conclusion:**

Our study shows that patients with a recurrent incisional hernia have significantly more subcutaneous and visceral adipose tissue than those with a primary incisional hernia. Further studies in this area are required if we are to reduce the burden of recurrent hernia following repair of a primary incisional hernia.

## Introduction

The prevalence of overweight and obese individuals is growing globally [1]. The association between obesity and the development of a primary or recurrent hernia is still controversial. It is general thought, however, that obesity is a risk factor for incisional hernia and may be implicated in the development of a recurrent hernia after a successful repair [2]. The mechanism for an increased risk of recurrent hernia in patients could range from technical factors, increased intra-abdominal pressure, abnormal distribution of fat or reduced muscle mass. Obesity may also have indirect effect on recurrence by increasing the risk of surgical site infection [3]. Muscle mass may also be affected by previous incisional hernia repair as a result of vascular or nerve damage from multiple repairs.

Body mass index (BMI) is often used as a proxy for obesity and has been considered a risk factor for the development of an incisional hernia. However, BMI is poorly associated with hernia development and does not accurately reflect fat distribution. Visceral obesity rather than BMI has been reported to be associated with greater incidence of wound complications and incisional hernia in patients undergoing colorectal surgery [4].

Computerised tomography (CT) is accurate method for quantifying measurement of regional skeletal muscle mass, visceral and subcutaneous adipose tissue [5]. NIH Image-J software of CT images has been used to quantify adipose tissue and skeletal muscle cross-sectional areas using standard Hounsfield units. Irving et al. used it to quantify total abdominal area, total fat area, subcutaneous fat area, visceral fat area and skeletal muscle area and they concluded that NIH Image-J is a reliable measurement of adipose tissue and skeletal muscle cross sectional areas [5].

The aim of this study was to examine the relationship between CT measured body adipose tissue and muscle area in patients with a primary and recurrent incisional hernia.

## Methods

A study of patients who had abdominal CT scan as part of their assessment for incisional hernia repair was undertaken. Patients were categorized into two groups: those with a ‘Primary’ or ‘Recurrent’ incisional hernia. Patients were obtained from a larger prospective cohort of patients who were being assessed for incisional hernia repair over a 2-year period Demographic data was collected prospectively on those patients while CT scans were reviewed retrospectively in the study cohort.

Patients’ height, weight, and ASA score were obtained from the prospective data base. CT image analysis using NIH Image-J software was undertaken for all patients. Image-J is a public domain, Java-based image processing program developed at the National Institutes of Health (NIH). NIH Image-J software is a free application available for everybody to download from the internet.

In our study, subcutaneous adipose tissue, visceral adipose tissue, and rectus muscle cross-sectional areas were measured at the level of L3/4 using standard Hounsfield unit ranges (adipose tissue: − 190 to − 30; skeletal muscle: − 29 to + 150).

**Fig. 1:**
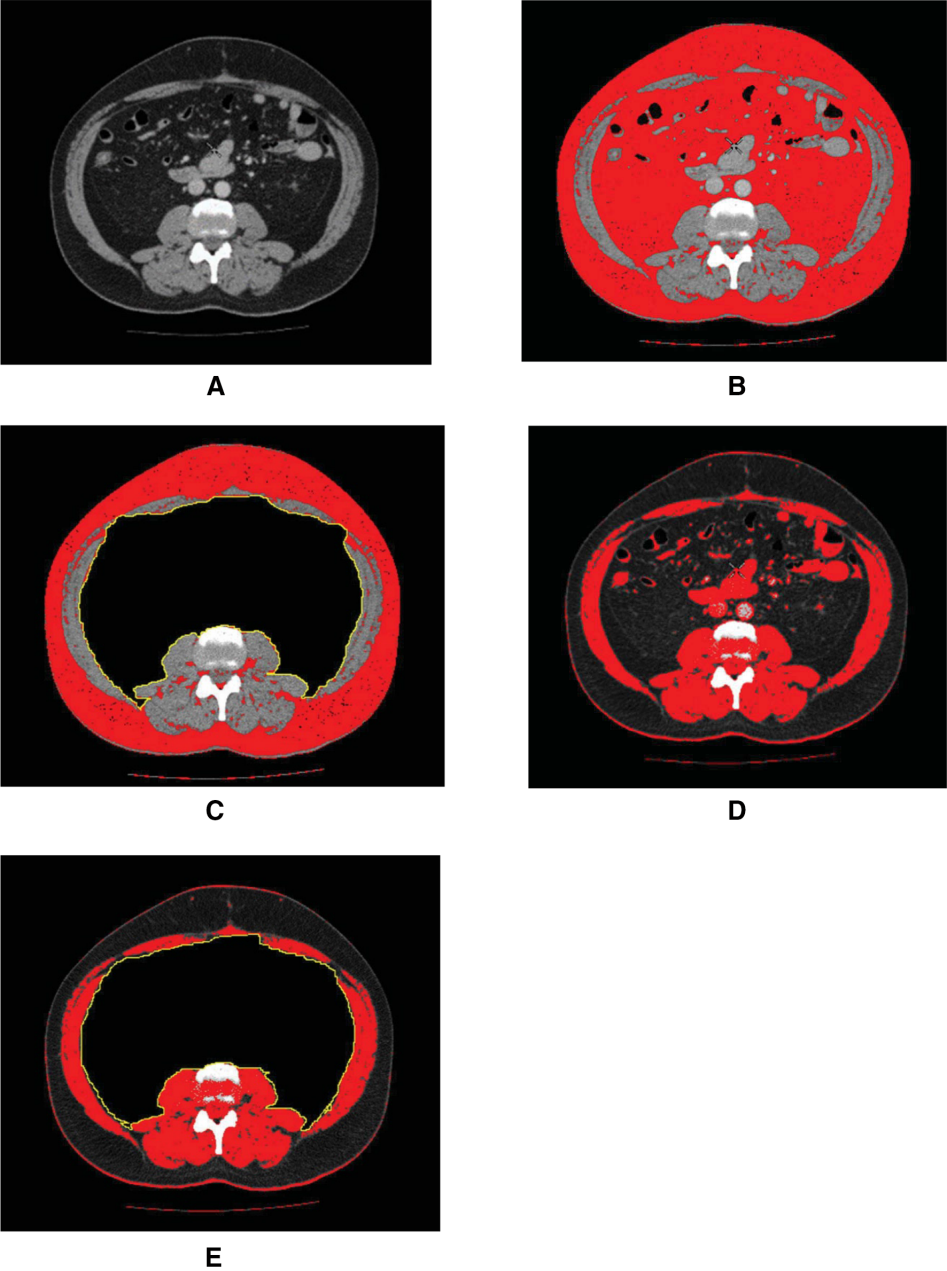
An example of calculating adipose tissue and muscle cross sectional area using J-image software. **a** Original CT **b** CT with adipose tissue thresholds applied—adipose tissues appear red. **c** Abdominal contents cropped, subcutaneous adipose tissue cross sectional area calculated. **d** Skeletal muscle thresholds applied, muscles appear red. **e** Abdominal contents cropped; total skeletal, left and right recti muscles cross sectional area calculated

The method and detailed steps for measuring ‘fat’ and ‘muscle’ cross-sectional area using Image-J software has previously been published and used in previous studies from our department [5–7]. Briefly, the original CT image at the level of L3/4 is converted to JPEG format. Adipose tissue thresholds (− 190 to − 30 Hounsfield Unit) are applied. The adipose tissue now appears in red colour. Visceral adipose tissue cross-sectional areas was measured first. Then the abdominal contents were cropped and the subcutaneous adipose tissue cross sectional area calculated. Skeletal muscle thresholds (− 29 to + 150 Hounsfield Unit HU) are applied. Muscles appear in red colour now. The abdominal contents were cropped and the total skeletal, then right and left rectus muscle cross sectional area calculated. This is illustrated in Fig. 1 in a patient with an incisional hernia in the epigastric region.

To test inter-observer ‘absolute agreement’, each parameter was measured independently by two investigators and reliability analysis performed. Additionally, right and left rectus muscles cross sectional areas were measured separately.

### Statistics

Age, weight, height and BMI were expressed by mean and range. Data distribution of CT scan measured areas was skewed and, therefore, data were considered non-parametric and expressed and analysed accordingly (Mann–Whitney test). For reliability analysis statistics, Interclass Correlation Coefficient test was performed. IBM SPSS Statistics for Windows, Version 22.0 (Armonk, New York, USA: IBM Corp) was used for analysis.

## Results

Thirty-six patients were included: 15 had a *Primary* incisional hernia while 21 had a *Recurrent* incisional hernia. The median number of operations for the recurrent group was 2 (range 1–14). The primary and recurrent groups were similar in age, weight, height, BMI, ASA grades and hernia defect width (Table 1). These patients were taken from a subset of 100 patients operated on during the 2 year period, 64 of whom did not have a CT scan. This group had a similar age range (23-85 years), sex distribution (55% female) and BMI (32, range 18-53). Although the percentage of female is higher in the study group this is not statistically significant (p = 0.881).

**Table 1:** Demographic data and characteristic of both groups

Variable	Primary group (*N* = 15)	Recurrent group (*N* = 21)	Difference between groups (*p*-value)
Age mean (range)	62 (24–82)	64 (41–82)	0.737
Sex (males: females)	6: 9	9: 12	0.869
ASA mean (range)	2 (2–3)	2 (1–3)	0.473
BMI mean (range)	31.4 (20–44.5)	31.9 (18.1 –43.6)	0.842
Weight-kg mean (range)	87 (51–130)	86 (47–110)	0.735
Height-cm mean (range)	163 (138–183)	166 (147–183)	0.446
Defect width-cm median (range)	7.5 (2–22)	9 (2.5–30)	0.751

Reliability analysis for CT-measured parameters showed very high Interclass Correlation Coefficient (ICC) between observers for subcutaneous adipose tissue (SAT) (0.993), visceral adipose tissue (VAT) (0.995), and skeletal muscle index (0.968).

Patients in the recurrent group had significantly larger cross-sectional areas of SAT (*p* = 0.042) and VAT (*p* = 0.033) than in the primary group (Table 2). Both groups did not differ in the cross-sectional areas for right (median = 2.8 cm^2^ vs 2.9 cm^2^) and left (median = 3.7 cm^2^ vs 4.1 cm^2^) rectus muscles (Table 2).

**Table 2:** Cross-sectional areas of adipose tissue and muscles for both groups

Cross-sectional area (cm^2^)	Primary group (*N* = 15)*	Recurrent group (*N* = 21)*	Difference between groups (*p*-value)
SAT	230.9 (164.7, 288.2)	321.9 (238.6, 348.8)	0.042
VAT	146.8 (120.0, 201.5)	221.1 (159.8, 300.1)	0.033
Right rectus muscle	2.9 (2.0, 4.4)	2.8 (2.0, 5.8)	0.704
Left rectus muscle	4.1 (1.8, 5.0)	3.7 (1.7, 6.3)	0.874

^*^Data are expressed as median and interquartile ranges

There was no difference in the total fat area (VAT and SAT) between men and women (495cm^2^ vs 500cm^2^, *p* = 0.936). The skeletal muscle area (both rectii muscles) was significantly greater in males than females (12cm^2^ vs 5cm^2^, *p* = 0.002).

## Discussion

This study shows that patients with a recurrent incisional hernia have significantly more subcutaneous and visceral adipose tissue on CT-scan when compared with a group with a primary incisional hernia. Both had similar BMIs and there was no difference in muscle mass between the groups. CT scan is considered the gold standard imaging modality for measurement of subcutaneous and visceral adipose tissue area [8]. CT is well suited to study the body fat because of its ability to distinguish readily between adipose and other tissues, due to its high contrast resolution. Finally CT scan is quick and easy to perform.

One of the difficulties in interpreting the findings from our study is what came first. Are patients with high subcutaneous and visceral adipose tissue levels more likely to develop a recurrent incisional hernia or are such levels a consequence of having a recurrent hernia?

A recent study by Winters and colleagues seems to confirm the former hypothesis [9]. They assessed factors involved in predicting complications in 65 patients undergoing complex hernia repair and found that visceral fat volume (VFV) was a significant predictor of recurrent herniation on multivariate analysis. In their study, VFV was measured by CT every 1.2 cm up to 12 cm cranially from S1. Reherniation occurred in 18 patients (27.7%) with an average follow-up of 14 months (range 0–82 months) [9].

A drawback of this study may be related to using only one CT slice for fat measurement. However visceral adipose tissue area as determined by a single CT scan between L3 and L4 level was shown to be constant and correlated to abdominal visceral fat mass obtained with a multiscan technique in the Framingham Heart Study [10]. They found that measurement of SAT and VAT at the L3/4 level correlated best with cardiometabolic risk factors for both men and women. The upper surface of L4 was the slice level we selected in our study because of the ease of reproducibly in identifying this location.

A further limitation of our work is the small number of patients involved in the study. It is clear that further research in this area is required. However, the findings from the Winters et al. and our study would seem to indicate that the volume of visceral and subcutaneous adipose tissue is likely to be a real factor in the development of a recurrent incisional hernia [9]. These findings occurred despite marked differences in patient groups, ours being significantly more obese and having a higher subcutaneous to visceral fat ratio.

Visceral adipose tissue is associated with greater obesity-related metabolic and cardiovascular disturbances and visceral fat is lost preferentially with modest weight loss [6]. This weight loss can be achieved by a very-low calorie diet and a supervised exercise program [6]. Patients may benefit from this intervention before operation for those capable of undertaking this task. The impact of such an intervention in obese patients with a recurrent incisional hernia may be beneficial but could only be proven by an appropriate designed clinical trial.

## Conclusions

Our study shows that patients with a recurrent incisional hernia have significantly more subcutaneous and visceral adipose tissue than those with a primary incisional hernia. This finding may indicate that such patients would benefit from either a very low-calorie diet and or an intensive exercise programme before operation. Further studies in this area are required if we are to reduce the burden of recurrent hernia following repair of a primary incisional hernia.

## Data Availability

Available if requested.
